# Comparative evaluation and co-relation in variation of curve of Spee and curve of Wilson in Class II div. 1, Class II div. 2, and Class III as against Class I malocclusion in central India population- an in vitro study

**DOI:** 10.12688/f1000research.133330.1

**Published:** 2023-05-15

**Authors:** Ruchika Pandey, Ranjit Kamble

**Affiliations:** 1 Postgraduate, Orthodontics, , Sharad Pawar Dental College and Hospital, Datta Meghe Institute of Higher Education and Research, Wardha, Maharashtra, 442001, India; 2 Professor, Orthodontics, Sharad Pawar Dental College and Hospital, Datta Meghe Institute of Higher Education and Research, Wardha, Maharashtra, 442001, India

**Keywords:** Curve of Spee, Curve of Wilson, Occlusion, Functional occlusion

## Abstract

**Introduction-** Getting acceptable cosmetic results in the soft tissues of the face serves as the foundation for orthodontic treatment planning. Also, in order to achieve healthy static and dynamic occlusal interactions, the teeth must be positioned within the basal bone at the correct position, angle, and inclination. To avoid periodontal issues, provide stability, and achieve a functional occlusion, it is essential to ascertain the individual’s dental arch form before starting of treatment and thus to utilise the mechanics that follow throughout the treatment.

**Objectives-** To evaluate and compare variation in Curve of Spee and Curve of Wilson in Class II Div.-1, Class II-Div-2 and Class-III as against Class I malocclusion in central India population.

**Methodology-** Irreversible hydrocolloid impression will be taken with perforated metal stock trays and stone cast will be poured. This will be scanned using CAD CAM machine and curve of Spee and Wilson will be measured using reverse engineering.

**Expected Result-** It will assist us in treatment planning for preventing periodontal issues, assuring stability, and achieving functional occlusion by evaluating and comparing the Spee and Wilson curves in Class II Divison-1, Class II Divison-2, and Class-III malocclusion with Class-I malocclusion.

**Conclusion-** Every single patient receiving orthodontic treatment has the COS, which is crucial to achieving a stable occlusion. Almost every patient who receives orthodontic treatment eventually experiences the Spee Curve. Since there aren’t many studies examining the relationship between the Curves of Spee and Wilson, their impact on dentoskeletal morphology, and their role in occlusal stability.

## Introduction

### Background and rationale

Planning for orthodontic treatment begins with achieving acceptable cosmetic outcomes in the soft tissues of the face. In order to achieve healthy static and dynamic occlusal interactions, it is also crucial to place the teeth in the basal bone with the correct angulation, inclination, and location. In order to prevent periodontal disease, provide stability, and achieve a functional occlusion, it is critical to identify the patient’s dental arch form before commencing treatment so that mechanics can be used to conform arch form throughout the therapy. Periodontal problems in teeth are caused by teeth moved orthodontically that go beyond the basal border and compromise the stability after treatment. ^1^ The occlusal plane should be flattened and the Wilson Curve should be levelled as the end results of orthodontic therapy. ^2^

The human dentition exhibits the natural phenomenon known as Curve of Spee (COS). For a successful masticatory system, the anteroposterior curve of occlusion is necessary. ^2^ In 1890, F. Graf von Spee (1855-1937), German anatomist, described the Curve of Spee. He discovered that the occlusion line in skulls with worn away teeth was a line on a cylinder perpendicular to the mandibular incisors’ incisal borders, surface of occlusal of 2 ^nd^ molar, and the anterior border of the condyle. ^3^ The COS, which is essential to attaining a stable occlusion, is present in every single patient undergoing orthodontic treatment. In orthodontics, the arc that is perpendicular to the incisal margins is currently referred to as the “Curve of Spee”. ^4^

Recent studies have shown that the mediolateral curve plays a biomechanical role in mastication by improving the efficacy of forces of occlusion during mastication and the crush/shear ratio between the posteriors. Dental malocclusions with significant overbites typically have an inflated Curve of Spee. ^3^ As a result, there will be an inappropriate functional occlusion since the muscle imbalance will be altered.

Wilson was the very first to distinguish the lower grinding teeth’s lingual inclination and the higher grinding teeth’s buccal inclination. The Curve of Wilson is the name given to this occlusal curve in the coronal plane. The Wilson Curve allows for lateral mandibular excursions without posterior obstructions. ^2^

For adequate function, the amount of buccolingual tooth inclination must be determined and quantified in order to support treatment objectives. ^2^ The palatal and buccal cusps of the posteriors are in functional contact as a result of this curvature, which appears to necessitate a concave mandibular arch as well as a concave and convex maxillary arch. ^5^ It would be logical to expect that the incline of bone would be oriented in this direction for the best masticatory stress given the alignment of the anatomical parts outlined by Dawson. Okeson outlined the purpose of the Curve of Wilson, which prevents balancing interferences and ensures the most efficient utilisation of cuspal contacts. ^2^

Andrews came up with 6 keys of normal occlusion out of which 3 ^rd^ key is tooth inclination, which is the labiolingual as well as buccolingual inclination of the crown. He saw a lingual tilt in the posterior crowns of the mandible and maxilla. ^2^ One of the elements included in the six keys to occlusion is the Curve of Spee. According to Andrews, COS in people with proper occlusion varies from flat to moderate. ^1^

The COS and COW in the lower arches of people living in central India will be determined in this study. In this study, reverse engineering is used to determine the Wilson curve and the Spee curve, which is useful for understanding its comprehensiveness as well as the impact it has on stable occlusion. The measurement and significance of compensatory curves have also been discussed in various studies.

In literatures prior to our study, some of the compensatory curves were shown to be correlated with dentoskeletal malocclusions and normal occlusion. The link between the Curves of Spee and Wilson and their effect on morphology of dentoskeletal, their potential to maintain stability of occlusion have all been the subject of a few studies. This investigation investigates the connection between compensatory curves and how it might aid in the development of treatment plans for specific malocclusions.

### Objectives

• To evaluate Curve of Spee and Curve of Wilson in Class II Div.-1, Class II Div.-2 and Class-III malocclusion in central India population.
• To evaluate Curve of Spee and Curve of Wilson in Class-I malocclusion in central India population.
• To compare variation in Curve of Spee and Curve of Wilson in Class II Div.-1, Class II Div.-2 malocclusion and Class-III as against Class I malocclusion in central India population.

**Trial design:** *In-vitro* study

## Protocol

### Study setting

Following study will be done at the Department of Orthodontics and Dentofacial Orthopedics, Sharad Pawar Dental College, Sawangi (M), Wardha, in cooperation with the Department of Prosthodontics.

### Eligibility criteria

Inclusion criteria:

• Patients of age group between 14-30 years.
• Complete permanent dentition except third molars considering it is the tooth that is most frequently lost owing to extraction or congenital tooth loss.

Exclusion criteria:

• Patient with previous orthodontic treatment.
• Patient with ongoing orthodontic treatment.
• Patient with missing or impacted teeth.
• Patient with systemic disease.
• Patient with previous record of trauma or surgery.
• Severe caries.
• Severe occlusal wear.

### Intervention

**Figure 1.**
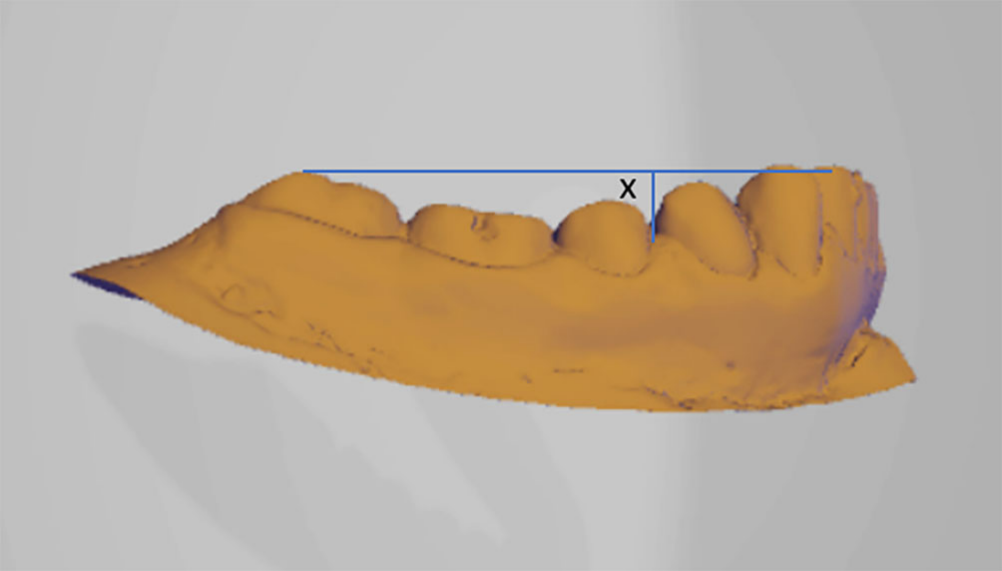
Curve of Spee will be taken from that tangent line to the deepest point on the premolar region.

**Figure 2.**
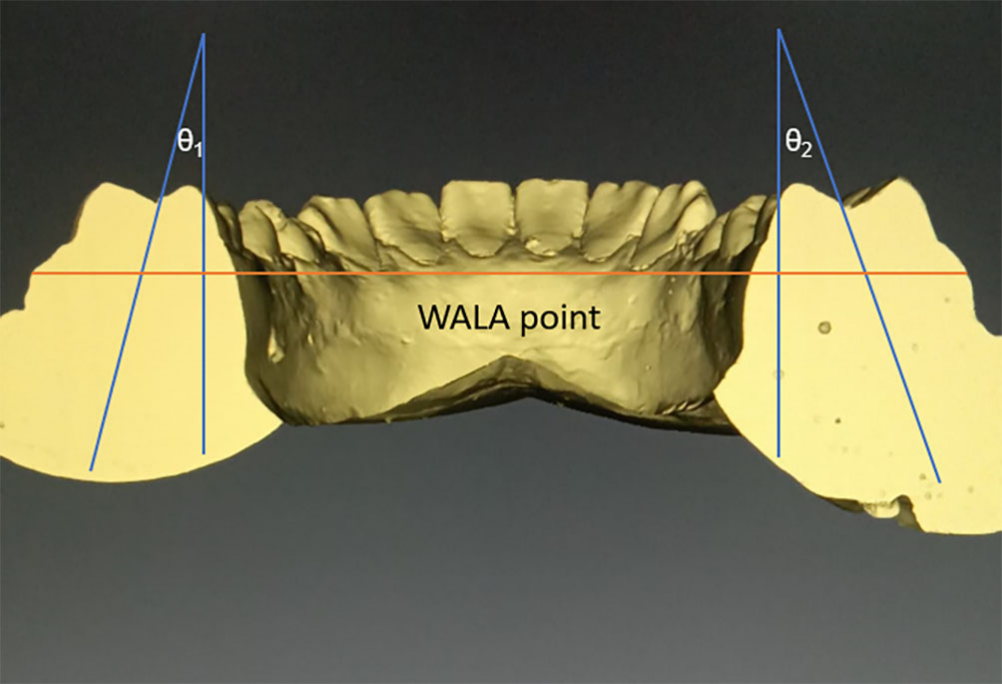
With WALA point used as a reference line Curve of Wilson will be measured.

• The study will be conducted on patients coming to the Department of Orthodontics and Dentofacial Orthopedics and diagnosed with Class I, Class ІІ div-1 and Class II div-2 & Class III malocclusion will be selected for the study.
• Written informed consent will be obtained from all the participants.
• Irreversible hydrocolloid impression will be taken with perforated metal stock trays and stone cast will be poured. This will be scanned using CAD CAM machine and curve of Spee and Wilson will be measured using reverse engineering. Reverse engineering is the practice of disassembling an object in order to discover how it functions. It is generally done to analyze and learn how something functions, although it is frequently used to copy or improve the end result.
• The curve of Spee will be measured by creating a tangent line connecting distobuccal cusp of mandibular 2 ^nd^ molars and highest tip of the mandibular incisors in a sagittal section. Measurements will be taken from that tangent line to the deepest point on the premolar region ( Figure 1). ^6^
• Frontal sections will be cut in the area of centre of 1 ^st^ molar. We shall follow the axis of molar, which follows a line that connects molars’ furcation and occlusal groove, to measure the Wilson curve’s angle. The greatest protuberance of the soft tissues present on buccal alveolar crest, the WALA points on the right and left bones, will be used to form a reference line. We can use it to calculate the angle between the left and right molars. The total angulation will be the sum of θ _1_ and θ _2_ ( Figure 2). ^5^

### Outcomes

To obtain a functional occlusion, it is essential to identify the patient’s dental arch form before the procedure and to apply mechanics that conform with the arch form throughout the therapy.

### Sample size

Formula for sample size when comparing two means

$$N=\frac{{\left(\mathrm{Z\alpha}+\mathrm{Z\beta}\right)}^{2}\left(\delta_{1}^{2}+\delta_{2}^{2}/k\right)}{\Delta^{2}}$$

Where;

*Z* *α* represents the degree of significance at 5% i.e., 95%

Confidence interval = 1.96

*Z* *β* is the test’s power = 80% = 0.84

*δ*_1_ = SD of COS depth in Class I = 0.717

*δ*_2_ = SD of COS depth in Class II = 0.732

∆=Distiction betweentwomeans=2.472–1.954=0.518

$$n=\frac{{\left(1.96+0.84\right)}^{2}\left(0.717^{2}+0.732^{2}/1\right)}{0.518^{2}}=30.67$$

*n* = 30 patients needed in each group

Total sample size is 120

Study Reference: IIknur Veli *et al*. ^7^

### Statistical method

All the results will be calculated using R version 4.2.3 (Shortstop Beagle **)**. Data for outcome variables will be tested for normality using Kalmogorov-Smirlov. Comparative analysis over the outcome of functional occlusion in different malocclusion will be evaluated and measurement of depth of curve of Spee and Wilson in millimeters respectively. ANOVA will be used to find significant difference in mean in comparison of 4 groups. Tukey test will be used for comparative evaluation of measurement in between 2 groups pairwise. P-value ≤ .05 will be considered significant 5% level of significance and 95% confidence of interval.

### Dissemination

This protocol will assist us in evaluating and comparing the Spee and Wilson curves in Class II Div-1, Class II Div-2, & Class III malocclusions as against Class-I malocclusions, & this will help us design our treatments in order to avoid periodontal issues, ensure stability, and achieve effective occlusion.

### Study status

Not started yet.

## Discussion

Dentists may use Spee curve analysis to predict how the occlusion will develop in the sagittal plane. The inclination of the masseter muscle was positively correlated with the Spee curve. The placement of the mandibular posterior teeth with this forward tilt increases the efficacy of the chewing muscles. In Wilson curve, the posterior teeth’s inclination is caused by two factors. The first has to do with loading resistance, and the second, with masticatory function. ^8^

Several of the compensatory curves have been linked to normal occlusion and dentoskeletal malocclusions in literature before our study. A few studies have looked at the relationship between the Curves of Spee and Wilson, their impact on dentition, and their capacity to preserve stability of occlusion. This study explores the relationship between compensatory curves and how it might help in formulating treatment regimens for particular malocclusions.

### Ethical considerations

Ethical approval received from Datta Meghe Institute of Higher Education and Research, Sawangi, Wardha

IEC reference number - DMIHER (DU)/IEC/2023/569

## Data Availability

No source data are associated with this article. Zenodo. Comparative evaluation and co-relation in variation of curve of Spee and curve of Wilson in class II div-1, class II div-2, and class III as against class-I malocclusion in central India population- an in vitro study. DOI: 10.5281/zenodo.7816557. File Name: SPIRIT_checklist RUCHIKA.docx Data are available under the terms of the Creative Commons Attribution 4.0 International license (CC-BY 4.0).
