# Supplementary material for: Comparative evaluation and co-relation in variation of curve of Spee and curve of Wilson in Class II div. 1, Class II div. 2, and Class III as against Class I malocclusion in central India population- an in vitro study
Source: F1000Res. 2024 Jul 10;12:493. Originally published 2023 May 15. [Version 3] doi: 10.12688/f1000research.133330.3 (PMC11211733; doi:10.12688/f1000research.133330.3)
Supplement: Peer review history

# Reviewer report: Reviewer response for version 2

**DOI:** 10.5256/f1000research.165137.r269001

**Published:** 2024-05-14

**Authors:** Liliana Ávila Maltagliati

**Affiliations:** 1 Guarulhos University, São Paulo, Brazil

This is a comparative study about curve of Spee and Curve of Wilson in malocclusion patients. It is unquestionable the importance of those curves to a healthy occlusion and it is interesting to determine if different malocclusions present different characteristics to be included in the treatment correction planning. Nevertheless some issues seems incomplete and in my opinion need revision:

 1. It was not clear if the group with class I malocclusion will be a control group or will be compared as a malocclusion group and so the research will have no control group. My question is based on the fact that class I occlusion not necessarily is a normal occlusion. Conceptually, class I is a malocclusion.

 2. Considering item 1, it would be a lot more interesting to have a group of normal occlusion as control group and then study curve of spee and curve of wilson deviation from normal occlusion

 3. The reference used to create methodology for curve of Wilson analysis use CBCT exams to achieve long axis of mandibular molar. Using cast models, it seems difficult to be sure about the exact localization of root furcation. I encourage authors to change curve of wilson determination methodology.

Is the study design appropriate for the research question?

Partly

Is the rationale for, and objectives of, the study clearly described?

Partly

Are sufficient details of the methods provided to allow replication by others?

Yes

Are the datasets clearly presented in a useable and accessible format?

Not applicable

Reviewer Expertise:

With methodological adjustments, the research may be resubmitted

I confirm that I have read this submission and believe that I have an appropriate level of expertise to state that I do not consider it to be of an acceptable scientific standard, for reasons outlined above.

# Response

**Published:** 2024-05-18

**Authors:** Ruchika Pandey

**Affiliations:** Orthodontics, Sharad Pawar Dental College, Datta Meghe Institute of Higher Education and Research, Wardha, Maharashtra, India

Thank you for thoroughly analyzing my article.

 1. It is apparent from the title and objective of this protocol that Class 1 malocclusion is used as the control group.

 2. Yes, it would have been better to use Class 1 normal occlusion as a control group rather than Class 1 malocclusion, but because they are uncommon and every person with Class 1 molar relation will have some type of malocclusion, it would be easier to use Class 1 Malocclusion as a control group.

 3. Yes, it will be difficult to find furcation in cast models, so it will be supported by OPG analysis, as performing CBCT on every patient will be costly and not available in every facility.

# Reviewer report: Reviewer response for version 2

**DOI:** 10.5256/f1000research.165137.r268428

**Published:** 2024-05-09

**Authors:** Luz Andrea Velandia Palacio

**Affiliations:** 1 CICO, Institución Universitaria Colegios de Colombia UNICOC, Bogotá, Colombia

The authors have addressed the suggestions made in the first review properly; I have no further comments to add.

Is the study design appropriate for the research question?

Yes

Is the rationale for, and objectives of, the study clearly described?

Partly

Are sufficient details of the methods provided to allow replication by others?

Yes

Are the datasets clearly presented in a useable and accessible format?

Not applicable

Reviewer Expertise:

Orthodontics and dentofacial orthopedics

I confirm that I have read this submission and believe that I have an appropriate level of expertise to confirm that it is of an acceptable scientific standard.

# Reviewer report: Reviewer response for version 1

**DOI:** 10.5256/f1000research.146309.r236704

**Published:** 2024-06-27

**Authors:** Vabitha Shetty, Dinesh Rao

**Affiliations:** 1 Department of Pediatric and Preventive Dentistry, AB Shetty Memorial Institute of Dental Sciences, Mangalore, Karnataka, India; 2 Peditric and Preventive Dentistry, AB Shetty Memorial Institute of Dental Sciences, Mangalore, Karnataka, India

An interesting study, especially useful in orthodontic rehabilitation of patients.Background, rationale and objectives are well written. The study protocol seems well thought about and structured. Sample size seems appropriate and satisfactory. Could the authors state the reasons they have chosen the CAD CAM machine for analysis of cast ? What are the advantages?

Is the study design appropriate for the research question?

Yes

Is the rationale for, and objectives of, the study clearly described?

Yes

Are sufficient details of the methods provided to allow replication by others?

Yes

Are the datasets clearly presented in a useable and accessible format?

Yes

Reviewer Expertise:

Sleep dentistry, preventive dentistry, cephalometric analysis

We confirm that we have read this submission and believe that we have an appropriate level of expertise to confirm that it is of an acceptable scientific standard.

# Response

**Published:** 2024-06-27

**Authors:** Ruchika Pandey

**Affiliations:** Orthodontics, Sharad Pawar Dental College, Datta Meghe Institute of Higher Education and Research, Wardha, Maharashtra, India

Thankyou Ma'am for thorough analysis of my article.

 Ma'am I am well aware that direct 3-D scanning intraorally produces a more precise result; however, due to the lack of an intraoral scanner in our location, we will have to scan the cast instead. Also, ma'am, we are employing the best CAD-CAM equipment, Dentsply Sirona. It has the essential feature of allowing you to work on machine-readable measurements and geometry, which is required for our investigation. Furthermore, it is highly precise, simple to copy, change, and annotate, and can be sent directly to milling for automated production. I can also send the STL file so that competent technical engineers can review the results and measurements.

 Thankyou

# Reviewer report: Reviewer response for version 1

**DOI:** 10.5256/f1000research.146309.r255515

**Published:** 2024-04-01

**Authors:** Luz Andrea Velandia Palacio

**Affiliations:** 1 CICO, Institución Universitaria Colegios de Colombia UNICOC, Bogotá, Colombia

The article underscores the significance of the Curve of Spee (COS) and Curve of Wilson (COW) in relation to occlusion and mastication, as well as their biomechanical functions. It further explores the correlation between these curves and dental malocclusions, their influence on treatment planning, and their connection to dentoskeletal morphology and occlusal stability. The aim of the study is to investigate the connection between compensatory curves and how it might aid in the development of treatment plans for specific malocclusions

 .

 I would like to point out the following in my review of this manuscript:  

1. The objectives are “to evaluate the curve of Spee and the curve of Wilson in Class II Div.-1, Class II Div.-2, and Class-III malocclusion in the central Indian population. To evaluate the curve of Spee and the curve of Wilson in Class-I malocclusion in the central Indian population.” corresponds to the steps followed in the procedure of the study, not the aims of the study. Perhaps it would be better to state that the study will identify the relationship between the Spee curve and the Wilson curve for each malocclusion type.
2. It is not clear if the measurements will be done by one or more observers; if so, it should be specified that an interrater reliability test will be performed and information regarding the observers experience in the field. 
3. Explain the lack of a direct 3D scan of the patients, and keep in mind the differences in accuracy between direct scanning and cast model scanning.
4.  In the Introduction section, where there is the following statement:” The occlusal plane should be flattened and the Wilson curve should be levelled as the end results of orthodontic herapy," reference number 2 is used incorrectly. The author who made that original statement corresponds to [1] Please correct.

Is the study design appropriate for the research question?

Yes

Is the rationale for, and objectives of, the study clearly described?

Partly

Are sufficient details of the methods provided to allow replication by others?

Yes

Are the datasets clearly presented in a useable and accessible format?

Not applicable

Reviewer Expertise:

Orthodontics and dentofacial orthopedics

I confirm that I have read this submission and believe that I have an appropriate level of expertise to confirm that it is of an acceptable scientific standard, however I have significant reservations, as outlined above.

## References

[1] : Orthodontics and Dentofacial Orthopedics. *J Orthod* .2002;29(2) : 10.1093/ortho/29.2.154-a 154 10.1093/ortho/29.2.154-a 28569105

# Response

**Published:** 2024-04-15

**Authors:** Ruchika Pandey

**Affiliations:** Orthodontics, Sharad Pawar Dental College, Datta Meghe Institute of Higher Education and Research, Wardha, Maharashtra, India

Thankyou sir for your thorough analysis of my article. I have made the following changes based on your review.

**1. The objectives are “to evaluate the curve of Spee and the curve of Wilson in Class II Div.-1, Class II Div.-2, and Class-III malocclusion in the central Indian population. To evaluate the curve of Spee and the curve of Wilson in Class-I malocclusion in the central Indian population.” corresponds to the steps followed in the procedure of the study, not the aims of the study. Perhaps it would be better to state that the study will identify the relationship between the Spee curve and the Wilson curve for each malocclusion type.**

**Response**- I have done the necessary changes.

**2. It is not clear if the measurements will be done by one or more observers; if so, it should be specified that an interrater reliability test will be performed and information regarding the observers experience in the field.**

**Response **-I have specified about interrater reliability test.

**3.Explain the lack of a direct 3D scan of the patients, and keep in mind the differences in accuracy between direct scanning and cast model scanning.**

**Response **- Regarding the lack of a direct 3-D scan of the patient, scanning intraorally will provide a more accurate result, however due to the unavailability of an intraoral scanner in our location, we will have to scan the cast instead. We will do our best to eliminate distortion while taking and pouring the impression cast.

**4.In the Introduction section, where there is the following statement:” The occlusal plane should be flattened and the Wilson curve should be levelled as the end results of orthodontic herapy," reference number 2 is used incorrectly. The author who made that original statement corresponds to [1] Please correct.​​​​​​​**

**Response **- I have done the correction.
